# Effects of a School Based Intervention on Children’s Physical Activity and Healthy Eating: A Mixed-Methods Study

**DOI:** 10.3390/ijerph16224320

**Published:** 2019-11-06

**Authors:** Matluba Khan, Ruth Bell

**Affiliations:** Institute of Health Equity, University College London, London, WC1E 6BT, UK; r.bell@ucl.ac.uk

**Keywords:** garden, children, physical activity, health behaviour, Green Gym, accelerometry, mixed methods, quasi-experiment

## Abstract

Combined diet and physical activity school-based interventions (rather than only diet or physical activity interventions) are more likely to help prevent children from becoming overweight in the long term. However, such interventions are less prevalent, and therefore, this pilot study aimed to assess the feasibility of a gardening intervention coupled with awareness about plant-based meals among 9−10 year old children in a London primary school. We recruited 60 children from two Year 5 classes, one class participated as an intervention group, and results were compared against another class who acted as the control group. Children’s physical activity (PA) was measured using GENEActiv wrist-worn accelerometers. Their fruit and vegetable intake and attitudes to and preferences in eating fruits and vegetables were measured using a self-report questionnaire. Furthermore, three focus groups were held with children in the intervention group to understand the reasons behind any change as a result of the intervention. Results are inconclusive; however, they indicate some impact on reduction of sedentary behaviour, increase of moderate to vigorous PA, knowledge of nutrition and some level of acceptance in trying new vegetables. School-based interventions involving gardening show some promise to increase children’s PA and improve their attitudes to eating fruits and vegetables.

## 1. Introduction

Childhood obesity is a major public health concern in the UK [1]. Today, 30% of children aged 2 to 15 in England are overweight or obese [2], and children who are becoming overweight or obese at earlier ages are likely to stay obese for longer [3]. Inequalities in child obesity have been increasing among children aged 10−11. The gap in obesity prevalence between the least and most deprived areas among 10−11 year olds increased by 5% between 2006/7 and 2017/18 [4].

The causes of obesity among children are complex and multifactorial, and a combination of measures is required to tackle them. Factors associated with overweight and obesity include unhealthy diet and insufficient physical activity [5,6]. There is evidence that a sufficient intake of fruits and vegetables is related to decreased risk of non-communicable diseases (NCD), including type-2 diabetes, cardiovascular disease and cancer [7,8]. Childhood is considered to be an important period for the development of healthy eating behaviours, including vegetable consumption, and children who adopt healthy eating behaviours at an early age continue to eat healthy diets into their adulthood [9,10]. The World Health Organisation (WHO) recommends eating at least 400 g or five portions of fruits and vegetables per day to reduce the risks of NCDs [11]. The European PRO-GREENS cross-sectional survey of 8158 eleven-year-old children from ten countries in Europe reported that the mean total fruit intake ranged between 114 and 240 g/d and vegetable intake between 73 and 141 g/d per day. The Health Behaviour in School-Aged Children (HBSC) study indicates that only 39% of 11 year old children consume vegetable and fruits on a daily basis, drawing on data from 44 countries in Europe and North America [12].

Again, benefits of regular physical activity (PA) for the current and future health of children and young people (5−17 years old) have been well researched and acknowledged by the World Health Organisation. WHO [13] recommends at least 60 minutes of moderate to vigorous physical activity for all children aged 5–17 years and the inclusion of vigorous and resistance activities at least three times a week. Yet, urban children are less active than the recommended levels in many countries, including the UK [14,15]. According to the HBSC study, only 50% of the children participated in two or more hours of vigorous physical activity per week [12]. Low vegetable and fruit intake and inadequate physical activity indicate the need for interventions for children that will encourage them to eat healthily and be more active on a regular basis.

Primary schools are typically the first formal institution where children spend most of their waking hours during term time. Hence, it is important that children have the opportunity to spend time outdoors and be active in this setting. The UK Government’s ‘Childhood Obesity: A Plan for Action: Chapter 2’ states “*We must ensure that schools are equipping children with the knowledge they need to lead healthy lifestyles and creating environments which encourage their pupils to eat healthily and be physically active (page 27)*” [16]. Again, physical activity and spending time outdoors is positively associated with mental health and academic performance [17,18,19]. Hence, a ‘whole-school approach’ would support children’s health and well-being.

Different school-based programmes introduced in the past decade to tackle childhood obesity focus on either dietary intake or improvement of physical activity. Interventions focusing on the promotion of healthy eating or improving physical activity have had limited effects on reducing childhood obesity. In contrast, complex interventions potentially addressing both diet and physical activity may show more promising results in tackling obesity [20,21,22]. The types of interventions included educational, environmental, and multicomponent, combining educational with environmental. The effect of school-based interventions including only an educational component (i.e., classroom-based activities) or only an environmental component (i.e., fruit and vegetable distribution) on children’s healthy eating is limited and not conclusive [23,24,25]. On the other hand, multicomponent interventions (including both educational and environmental components), show more promising results in increasing children’s fruit and vegetable consumption [20,23,24]. 

The experiential learning approach taken in this study by setting up a school garden and creating awareness about eating healthily, incorporates both the environmental and educational components addressing both dietary intake and physical activity. The health and well-being impacts of school gardens on children’s health and well-being are reported in the systematic review conducted by Ohly and colleagues [26]. School gardens can positively influence children’s vegetable and fruit intake [27,28,29] and their physical activity [30,31]. However, in many of these interventions, the gardening activities were not linked with their meals. Knowledge of nutrition and the reference daily intake and self-efficacy have been found to be positively associated with fruit and vegetable intake [32]. The benefits of school gardening can be amplified by incorporating hands-on learning of growing fruits and vegetables with curricular learning and school meals, making a connection between what children eat with what they could grow in their school gardens. 

This study was conducted within the framework of INHERIT. (INHERIT is a Horizon 2020 project aiming at identifying and implementing policies/practices/innovations that promote health, reduce health inequalities and improve the environment). This study aimed to increase children’s physical activity and improve their attitudes to healthy eating by combining activities in the school gardens with provision of a plant-based meal once a week. The activities in the garden were run by a Schools and Community Education Project Officer (SCEO) from the Green Gym with support from class teachers, and resources from the Meat-Free Monday UK campaign. The Green Gym ® is a group outdoor activity offered by The Conservation Volunteers (TCV) to help people get physically active and make a difference to the local environment with an emphasis on health and fitness. Anyone can join free outdoor sessions where volunteers are guided in practical activities such as planting trees, sowing meadows and establishing wildlife ponds.), The Meat Free Monday campaign aims to raise awareness of the detrimental environmental impact of animal agriculture and industrial fishing, and encourages people to slow climate change and improve their health by having at least one plant-based day each week. The study was approved by the University College London Research Ethics Committee and consent was gained from both parents and children before the study commenced. The project identification code is 12543/001, date of approval is 3rd July 2018.

The ‘INHERIT model’ [33] underpinned the conceptual framework for this research study, and is a relational model built on concepts used in the long-established DPSEEA (Drivers, Pressure, State, Exposure, Effect, Actions) model [34] and behaviour change wheel (BCW) [35]. The INHERIT Model comprises interconnected components and offers the basis for design, planning and evaluation of INHERIT case studies/interventions to frame, describe and assess the relationship between environment, human health and well-being and other factors. The model further facilitates the understanding of how interventions and actions can affect lifestyle behaviours by showing the causal pathway.

In the present study, an intervention is designed to change behaviour by offering opportunities to participate in gardening activities, to build capacity by learning about gardening and plant-based healthy diets, and to increase motivation amongst both teachers and children by incorporating these activities into regular curricular lessons of the school. The intervention thus enables three essential conditions: capability, opportunity, and motivation (what is termed as the ’COM-B system’ forming the hub of the BCW) [35]. The intervention was carried out in a public primary school in North-East London in the United Kingdom. Using a pre-post design the study investigated the impact of the above-mentioned activities (gardening with provision of plant-based meals once a week) on children’s physical activity and the key determinants of fruit and vegetable intake (FVI), i.e., attitudes to and preferences in healthy eating and knowledge of nutrition and plant science. An intervention group (IG) and a control group (CG) were selected in the same school, where the former received the intervention and was compared against the CG, who were not exposed to gardening activities during the period of the experiment. The following hypotheses were examined quantitatively:

**Hypothesis** **1:** The intervention group (IG) would report significantly less sedentary behaviour (SB) and more moderate to vigorous physical activity (MVPA) compared with the control group (CG).

**Hypothesis** **2:** The IG would report significantly more daily consumption of fruits and vegetables than the CG. Significant difference is also predicted between the two groups’ attitudes to and preferences in eating fruits and vegetables.

**Hypothesis** **3:** The IG would have significantly better knowledge of nutrition and plant science compared with the CG.

Qualitative methods were used to understand the underlying reasons behind any difference between the two groups and how the intervention might have contributed to any change in the intervention group.

## 2. Materials and Methods 

A quasi-experimental mixed methods study was conducted in a public primary school in the London Borough of Redbridge in North-East London. The borough is diverse in its profile with 11 neighbourhoods amongst the 20% most deprived in England and another 11 amongst the 20% least deprived in England [36]. The borough is the 21st most deprived out of 33 local authorities in London, and 15.5% of children in this borough come from low-income families. The children in the school also come from diverse backgrounds, where 4.2% come from the 10% most deprived families of the borough, and 1.3% come from the 10% least deprived families of the borough. 12.3% of children are eligible for free school meals (see Table 1).

**Figure 1:**
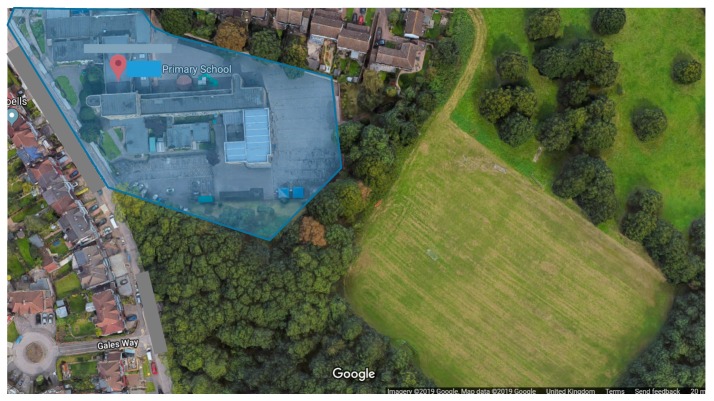
Google earth image of the school showing the school premises and the surrounding area.

**Figure 2:**
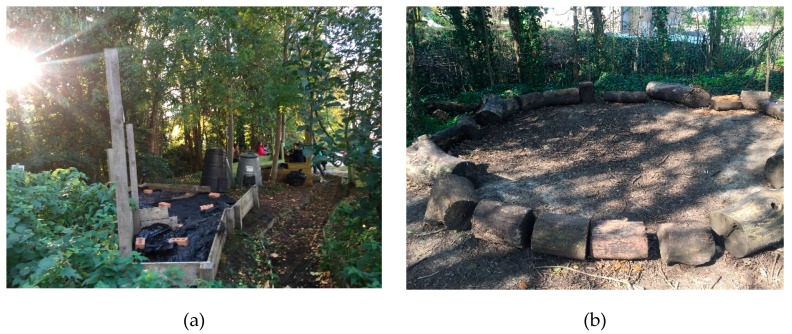
(**a**) The mound area with raised beds and the compost bins, (**b**) An outdoor seating area next to the trail.

In terms of the physical environment of the school, there is a tarmac playground with a playhouse, a shaded area with picnic tables, some seating areas, planters and a mound area. The mound area has 7 raised garden beds of different sizes, 2 compost bins, a small seating area and a small pond (see Figure 1 and Figure 2a). The school also has access to a green field and woodland adjacent to the school premises; however, this area is not used on a regular basis. There is an outdoor classroom/seating area along a trail in the woodland (see Figure 2b). Before intervention, the mound area was overgrown with weeds and the children were not engaged in any outdoor learning sessions there.

### 2.1. Study Participants

Sixty children (9−10 years old) from Year 5 participated in the study, where 30 children in one class were the intervention group (IG) and the other parallel class of 30 children acted as the control group (CG) (random group assignment). Children had been randomly allocated by the school to these two classes at the beginning of the school year. Both groups included children with different learning abilities and included children with autism and hearing impairment. Children aged 9−10 were selected for two reasons. Firstly, the research methods used in this study would be developmentally appropriate for children of this age (i.e., questionnaires and focus groups), and secondly, the obesity rate among Year 6 children (10−11 years old) in England is of public health concern [4]. Therefore, identifying potential routes to obesity and overweight management among children closer to this age is crucial. 

### 2.2. Intervention

The intervention was a collaboration between the UCL Institute of Health Equity, TCV and the Meat-Free Monday Campaign UK (MFM), and included leading children outdoors for activities related to gardening, growing of food and environmental improvement and conservation every Monday afternoon during the school term for two hours. The intervention was planned to run for one school year, where the intervention group (IG) had access to the gardening activities outdoors run by the TCV for the first half of the year (September 2018 to February 2019) when the control group (CG) received their usual classes indoors. They (IG) also received one Meat-Free Monday session run by the MFM UK campaign manager (30 min), focusing on the environmental and health benefits of plant-based meals. The activities focused on encouraging children to eat more vegetables and fruits, and taste and try new vegetables; they were also encouraged to eat a plant-based meal at least once a week. For the second half of the year (February 2019 to July 2019), the CG children would have access to gardening activities and one Meat-Free Monday session. This paper reports results based on the data collected after five months of intervention in February 2019. TCV led the outdoor activities based on their experiences and guided by evidence generated from previous Green Gym evaluation studies [37]. The UCL research team facilitated the activities, liaising with the school and ensuring access to resources needed for the intervention, and assessed the feasibility and impact of the intervention.

**Figure 3:**
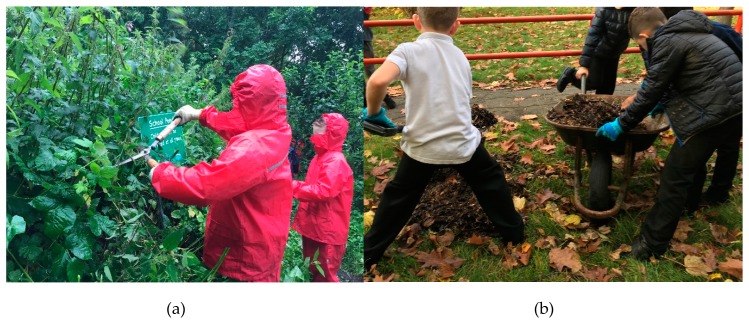
(**a**,**b**) Some examples of physical activities children were engaged in during gardening.

On the first day of gardening sessions, the SCEO introduced herself as a facilitator of the gardening activities rather than the leader. During the first few weeks, children mapped the outdoor area and suggested changes and improvements that could be made to the school ground. The Green Gym activities in the school ground were then based around the suggestions made by children that included preparing raised beds for spring crop growing by weeding, covering and refilling with newly dug leaf mulch and compost from school grounds (Figure 3b). Children also sowed seeds of cress and lettuce in toilet rolls in their green house to transport later on to the garden beds. Children worked on creating a dead hedge as a safety barrier by collecting, sawing, hammering and weaving the wood themselves. They worked together to use slabs to create an accessible path to the garden shed, cleared the field path, removed small trees and relocated the mini-beast (insect) hotel and the compost bin. Children learned how to light small fires with no matches or lighter, collected twigs for fuel and lit kettles to heat the water for a festive hot chocolate treat before Christmas. They also tasted a wide variety of fruits and vegetables during one session.

### 2.3. Outcome Measures

Baseline assessments (before the intervention) were carried out at the beginning of Year 5, a week before the gardening session started. The outcome assessment was completed immediately after the intervention (middle of Year 5). Identical protocols and procedures were used at both assessments. They were undertaken by trained researchers who had completed enhanced Criminal Records Bureau/Disclosure and Barring Service checks. 

Children were asked to wear a GENEActiv accelerometer (GAwrist, Activinsights, Cambs, UK) on the non-dominant wrist for seven consecutive days. The instruction was to wear the devices at all times including during sleep and water-based activities. Devices were set to record at a frequency of 100 Hz.

A self-reported questionnaire was used to measure children’s attitudes to, frequency of and preferences in eating fruits and vegetables. This reliable and valid questionnaire was developed to assess dietary patterns associated with positive energy balance and food behaviours, attitudes, knowledge and environments associated with healthy eating among Year 5, 6 and 7 children [38]. As the current study only assessed attitudes to, frequency of and preferences in fruit and vegetable consumption, only these questions were kept in the questionnaire. The questionnaire further included items to measure children’s knowledge of plant science and nutrition used by Wells et al. [39]. The children completed the questionnaires in their regular classroom environment. Children were given instructions on how to complete the questionnaires to ensure sufficient understanding. For questionnaire items and the response scale see Table 2.

### 2.4. Qualitative Method

In order to gain insight into the underlying reasons behind any change due to intervention and the children’s experiences of gardening, what worked and what did not, qualitative information was sought through three focus groups with children after the intervention. Each focus group comprised four to six participants. The focus group discussion (FGD) was semi-structured and explored topics around the children’s experiences of gardening outdoors, whether gardening helped or deterred their learning and whether or how gardening had any positive/negative impact on their behaviour, physical activity and attitudes to eating fruits and vegetable. FGDs also explored children’s views of how the activities can be improved.

Teachers and instructors from Green Gyms responded to a set of open-ended structured questions in written format. The questionnaire included ten questions asking about their experiences of gardening, what went well and what did not go well and how the intervention could be improved further.

### 2.5. Data Analysis

GENEActiv wrist data were downloaded using the GENEActiv software version 3.2 and saved as binary files. Files were then processed in R following van Hees et al. [40]. Days with ≥10 hours of wear time were considered valid. Sleep time was considered as the hours between midnight and 06:00 and was excluded from analyses. Children with at least 3 valid days were included in analyses. Data were segmented into the whole day from 06:00 until midnight and school time from 09:00 and 15:00 on weekdays. Daily averages were then calculated for each activity threshold across the whole day (06:00 to 24:00 H) and during school time (SH: 09:00 to 15:00). The data from the first day of wearing the devices was excluded because of potential reactivity to the measuring equipment, while the remaining wearing days were checked for validity.

Prior to all analyses, all outcome measures were checked for normal distribution (skewness and kurtosis between −2 to 2). The data from all the children from two groups have been explored together on each of the variables. All outcome measures were normally distributed. Descriptive statistics (using SPSS 24.0 for Windows (IBM Corp., Armonk, NY, USA)) were computed to describe the sample characteristics. Independent sample t-tests were conducted at baseline (T1) to assess whether there was any difference between the two groups at baseline. To assess the effectiveness of the intervention, parametric tests (one-way ANCOVA) were selected to compare the groups. In addition, a parallel samples t-test was conducted for the intervention group to measure any improvement between baseline (T1) and follow-up (T2). The data from the focus groups and structured questionnaires with teachers were analysed using thematic analysis. Qualitative data analysis software Quirkos 2.4 (Quirkos Software, Edinburgh, UK) was used for the analysis. 

## 3. Results

### 3.1. Baseline Measures

The mean age of the sample was 8.92 years (range between 8 and 10 years old) and 39% were girls. There was no significant difference between the treatment group and the control group in sample characteristics in terms of age and sex. However, significant differences were found between the two groups in sedentary behaviour during school hours and daily fruit consumption, with the IG scoring high at sedentary behaviour (900−1500) (t(36) = 2.110, *p* = 0.042) and the CG scoring high at light physical activity (LPA) (t(36) = −2.216, *p* = 0.033) and daily fruit consumption (t(55) = −2.481, *p* = 0.02). The baseline measures can be found in Table 3.

### 3.2. Intervention Effects

There was no significant difference between the two groups at the follow-up after five months of intervention using an independent sample t-test. In a parallel sample t-test, no significant improvement was measured for the intervention group at T2 compared to T1.

### 3.3. Intervention Effects Taking into Account Baseline Scores

In a one-way ANCOVA controlling for baseline scores, there was no significant difference between the intervention and the treatment group in any of the outcome measures (see Table 4). However, the mean for some measures (fruit consumption and knowledge of nutrition and plant science) for the intervention group indicates a positive trend compared to the control group. Individual measures are discussed in further detail below.

#### 3.3.1. Sedentary Behaviour

**Figure 4:**
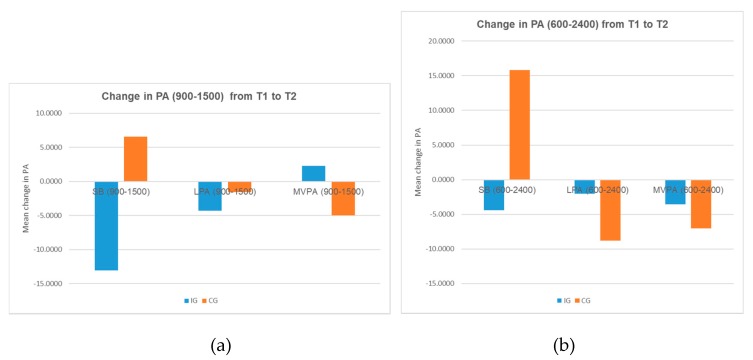
(**a**) Change in physical activity during school hours over time. (**b**) Change in physical activity during waking hours over time.

In all groups at both baseline and follow-up, mean sedentary time represented more than half of the school day (65%−71%). At baseline, the intervention group spent significantly more time in SB than the control group during school hours. Post-intervention, the difference between the two groups at SB measures was not significant. Times in SB reduced for the intervention group, whereas they increased for the control group. Accounting for the baseline scores, there was no significant difference between the groups because of the intervention (*p* = 0.592). The change in SB measures during school hours over time (T1 to T2) between the two groups was not significant (*p* = 0.449); however, the trend is opposite for the two groups (Figure 4a), which indicates some effects on reduction of times spent being sedentary among the IG because of the intervention.

The average daily sedentary time (06:00−24:00) represented more than two-thirds of the waking hours for both groups at baseline and follow-up (76−81%). Post intervention, there was no significant difference between the groups accounting for the baseline scores (*p* = 0.327). However, times spent being sedentary decreased for the IG, whereas it increased for the CG. Although the change in daily SB measures during waking hours over time (T1 to T2) between the two groups is not significant (*p* = 0.207), the trend is opposite for the two groups (Figure 4b), which indicates some effects on the reduction of daily sedentary times among the IG because of the intervention.

#### 3.3.2. Physical Activity

Both at baseline and follow-up, all the groups spent more time in LPA and less in MVPA during school hours. Post intervention, the difference between the groups in LPA and MVPA measures is not significant taking into account the baseline scores. The difference in change in LPA and MVPA measures over time during school hours between the two groups is not significant; however, the IG experienced a greater decrease in LPA but an increase in MVPA. The trend for the CG was the opposite (Figure 4a and Table 5).

**Figure 5:**
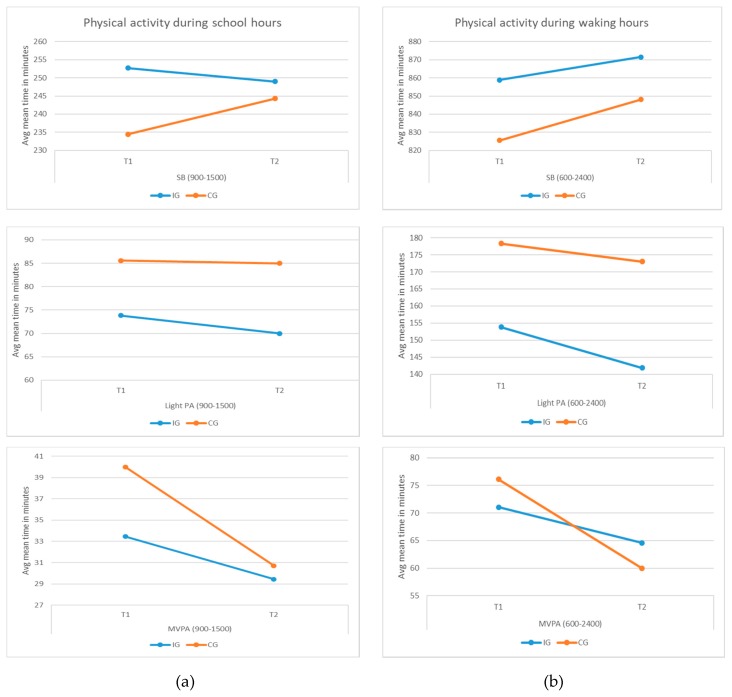
(**a**) Physical activity (in minutes) during school hours (09:00−15:00). (**b**) Physical activity (in minutes) during waking hours (06:00−24:00).

The average daily time spent on LPA and MVPA reduced for both groups after the intervention. Taking into account the baseline scores, the difference between the two groups was not significant. The difference in change from baseline to post intervention in LPA and MVPA measures during waking hours between the two groups was not significant. Although the reduction in LPA among the IG was similar or more than the CG, the reduction in MVPA for the IG was much less compared to the CG (Figure 5b). Hence, the results indicate some positive changes in time spent on MVPA because of the intervention.

#### 3.3.3. Daily Consumption of Vegetables and Fruits

**Figure 6:**
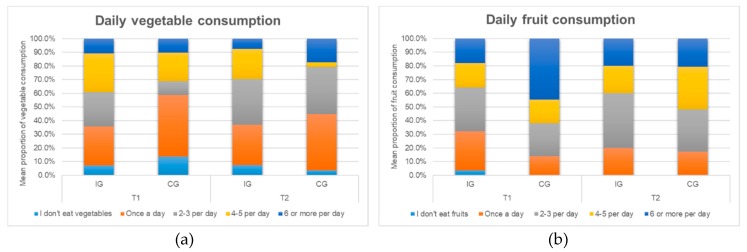
(**a**) Daily vegetable consumption of children before and after intervention. (**b**) Daily fruit consumption of children before and after intervention.

At baseline, 7.1% of children from the intervention group and 13.8% of children from the control group reported that they did not eat any vegetables. Post intervention, this number did not change much for the intervention group, while only 3.4% of children from the control group reported that they did not eat any vegetables. There was an increase in the percentage of IG children eating one serving (28.6% to 29.6%) and 2−3 servings of vegetables per day (from 25.0% to 33.3%), on the other hand, there was a decrease in the number of children who ate 4−5 servings a day (28.6% to 22.2%) and 6 or more servings a day (10.7% to 7.4%). In contrast, an increase was observed in all cases (except 4−5 servings) for the CG children (see Figure 6).

**Figure 7:**
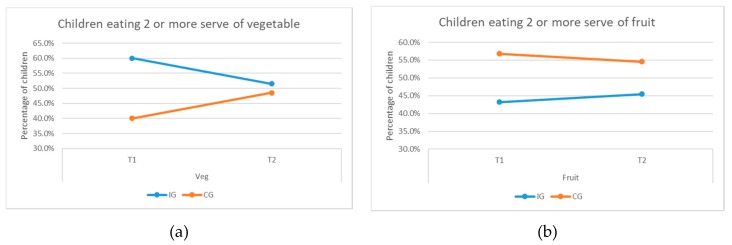
Percentage of children eating two or more servings of (**a**) vegetables and (**b**) fruits.

There was an increase in the IG children’s consumption of fruits after the intervention, although this was not statistically significant (there was a significant difference between the two groups at baseline, the CG scored higher). At baseline, 3.6% of the IG children had reported not eating a single serving of fruit, the percentage was zero post intervention (see Figure 6b). The percentage of children eating more than two servings of vegetables decreased for the IG children (see Figure 7a); however, the percentage of children eating more than two serving of fruits increased for them (see Figure 7b). The results indicate some effects of the intervention on children’s consumption of fruits but not vegetables.

#### 3.3.4. Attitude to Eating Vegetables and Fruits

**Figure 8:**
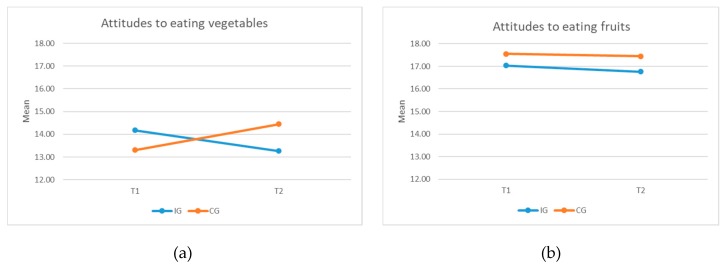
Attitude to eating (**a**) vegetables and (**b**) fruits.

Post intervention, no significant difference was observed between the two groups in their attitude to eating vegetables and fruits, taking into account the baseline scores. The change among the two groups from T1 to T2 was not pronounced (see Figure 8b). However, the mean for attitude to eating vegetables was higher for the CG children after the intervention, while it was the other way around before the intervention (see Figure 8a).

#### 3.3.5. Preferences in Eating Vegetables and Fruits

**Figure 9:**
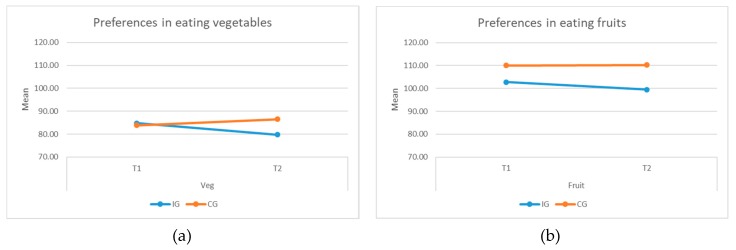
Preferences in eating (**a**) vegetables and (**b**) fruits.

**Figure 10:**
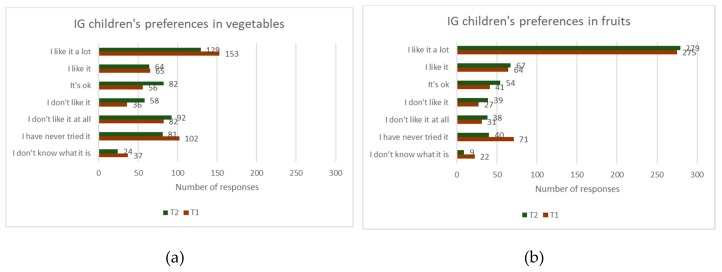
Intervention Group (IG) children’s preferences for (**a**) vegetables (**b**) fruits, before and after intervention.

No significant difference was found among the groups in their preferences in fruits and vegetables. A slight decreasing trend in preferences for fruits and for vegetables was observed for the IG children (see Figure 9a,b). However, the IG children showed some levels of improvement in knowing new vegetables and fruits and making an attempt to try something new. Out of 531 counts of different preferences to vegetables, 24 were recorded as ‘don’t know what it is’ and 81 were marked as ‘never tried it’ at T2, in contrast to 37 and 102 at T1 (see Figure 10a). For fruits, among the IG children, 9 and 40 responses were recorded as ‘don’t know what it is’ and ‘never tried before’ respectively, at T2, in contrast 22 and 71 were recorded at T1 (see Figure 10b).

#### 3.3.6. Knowledge of Nutrition and Plant Science

**Figure 11:**
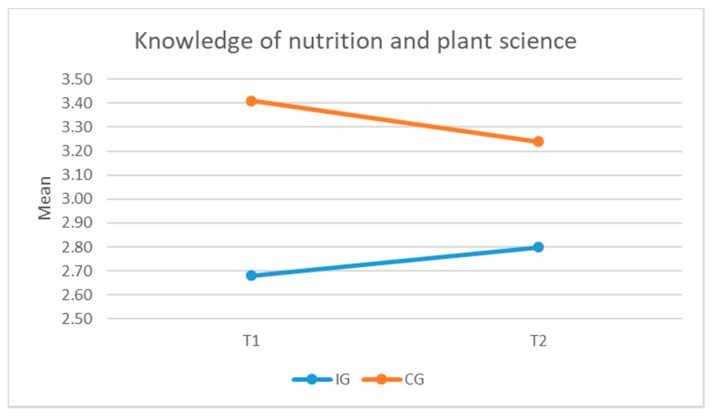
Difference in children’s knowledge of nutrition between groups, before and after intervention.

No significant difference was found between the two groups in their reported knowledge of nutrition of plant science both before and after intervention. However, the mean for the IG children was higher at T2 than T1, in contrast to the CG children whose mean at T2 was lower than T1. Although not significant, this indicates some improvement in the IG children’s knowledge of nutrition and plant science (see Figure 11).

### 3.4. Qualitative Insights

The findings from the post-intervention focus group discussion with children and interviews with teachers are discussed around the following three themes: healthy eating, physical activity and sociality.

#### 3.4.1. Healthy Eating

In general, the intervention group children reported a change in their attitudes towards eating vegetables. Most children mentioned that they ate fruits on a regular basis, but because of the intervention, they were now eager to try vegetables even if sometimes they thought they did not like the vegetables, as evidenced in the following conversation:

Jack (All names used in the reporting of qualitative results are pseudonyms.): When like green gym wasn’t in our school I wasn’t really keen on vegetables, I wasn’t keen on like, whenever I had a meal at a restaurant I’d be like ‘oh mum can you eat my peas please cos I don’t want them?’

Interviewer: Yeah

Jack: But now I’m like, my mum asks ‘shall I take your peas?’ and I’m like ‘no I’m fine’

Also, here:

“Before green gyms started I used to love fruit but hate vegetables. And so, every time my mum put vegetables on my plate and I had something else with it, I’d just eat the other thing but then just leave all the vegetables away, but now if I look at them I won’t throw them away, I’d eat every single thing that’s on the plate and I wouldn’t moan about it”

Some children also reported that there was not any change in their attitude to eating fruits and vegetables, whereas some others mentioned that they would have tried new vegetables had they had the opportunity to plant them.

*“I don’t try food that much, but if I actually plant it, it might actually kinda make me try it.”*

*“Imagine if you plant your own food and you taste it, and you think ‘oh that’s really nice’ and eat more of that and eat less of like chocolate.”*

Some of them also mentioned about their dissatisfaction about the meat-free options in the school menu.

*“I think when Meat-Free Monday came in I actually tried to do Meat-Free Monday sometimes, but it’s not going very well, because lunches on Monday is always meat, so there was sausages. I was going to go for the vegetarian ones, but I tried that last time and they were not very nice.”*

#### 3.4.2. Physical Activity

Children reported that they were more active in the school on Mondays than before the intervention. They also mentioned that they grew muscles as they were engaged in different kinds of gardening activities, as evidenced here:

“Because I’ve been walking around a lot and running so my legs are gaining muscle and my arms they’re always moving like chopping, raking or using shovels, and sometimes with the shovels there’s parts that are really hard but I push on and I’m able to get it up.”

Children also reported that they were eager to explore more outdoors as they started spending time outdoors with Green Gym.

*“Cos I live in a house and I have a dog we usually just take her out in the garden but since green gym like when I get home from school ‘I’m like mum can we go walk the dog now?’ and she’d be like ‘yeah one minute’ and before I wouldn’t ask to walk the dog.”*

#### 3.4.3. Sociality

Children reported that working together in gardening groups helped them connect with others, get to know each other better and make new friends. Children expanded further on how gardening helps:

“Basically you can be put in groups that you don’t really like people and then like, you kind of get to know them, and like, you becoming friends with them, so it’s helping you in a way.”

They also thought nature can help friends come closer even after an argument, as mentioned here:

“I feel like nature and plants and stuff bring people together. Because I remember, me and my friend had an argument. And then during Green Gym, we kind of slowly came back together.”

Focus group discussions also reflected that some children were kinder and more helpful to peers during outdoor gardening sessions, whereas they were naughty in the classroom.

*“Some people they’re naughty in class, but some people, when they went out there they were like helping everybody. Because there was someone who quickly learnt how to do it and he kept helping a bunch of people because he understanded (understood) and he wasn’t really playing around, and he was like sticking and understanding.”*

## 4. Discussion

The present study investigated the impact of a gardening intervention aided with a Meat-Free Monday session on children’s healthy eating, physical activity and knowledge of nutrition. Results suggest that school gardens help to reduce children’s sedentary activity and promote PA. The analysis of data collected using accelerometers indicated that compared to children not engaging in any gardening activities, children having a weekly two hour session of gardening reported a greater reduction (though not significant) in their usual daily sedentary activity during the school hours. Though measures of MVPA during school hours and waking hours reduced for both groups, this reduction was less among children engaged in gardening activities, indicating a positive impact on MVPA due to intervention; however, a causal connection should be made with caution as the difference is not significant. Though there is little prior research examining the effects of school-based gardening interventions on PA, our findings are consistent with the previous study by Wells et al. [30]. 

The qualitative findings from the study indicate a positive impact on children’s physical activity, children reported being more active than before, building muscles and improving their gross motor skills. This aligns with findings from previous studies [30,31] that reported that children moved more and sat less on days when they were gardening.

Regarding healthy eating, the findings of the study indicated no effects on children’s vegetable consumption and attitudes to eating vegetables, although the existing literature indicated that school-based complex interventions and experiential learning approaches are more effective in influencing children’s healthy eating [24,41,42,43]. The finding from this study resonates with the findings from a recent study by Huys and colleagues [44]; however, it contrasts with the findings from the above-mentioned studies [24,42,43]. The findings from the quantitative analysis also did not indicate any significant impact on fruit consumption, attitudes to and their preferences in eating fruits. However, children have shown some improvement in their familiarity with new vegetables and fruits and interest in tasting new ones. 

The focus groups with children, however, indicated that they were more motivated to taste vegetables, that in many cases they thought it was acceptable to eat some vegetables, and that the taste was somewhat acceptable in more cases after intervention than before. It was also indicated in the focus group conversations with children that if they had the opportunity to grow fruit and vegetables, and taste them in the school gardens, that might have had an impact. This resonates with the findings from the qualitative study by Sarti and colleagues [45], who explored children’s perspectives on school gardening and vegetable consumption, where children stated that they ate vegetables because they had grown them with their own hands.

Regarding knowledge about nutrition and plant science, findings from this study indicate some improvement in children’s knowledge of nutrition and plant science because of the intervention, although the difference is not significant. The positive impact on children’s knowledge is harmonious with previous studies [27,39,46]. The qualitative findings also indicated more opportunities for social interactions and making friends with other pupils.

### 4.1. Interpretation

It is not surprising that findings from the accelerometer data did not reveal statistically significant effects considering the relatively short five month intervention period and a small sample size. Although school gardening is expected to activate children to do activities outside school hours, it seems unlikely to happen within the short timeframe of this study. In addition, the post intervention data were collected in winter, when people usually spend more sedentary time because of shorter days and longer nights. Reduction of sedentary behaviours is associated with decreases in percentage overweight and obesity and decreased risk of cardio metabolic diseases [47,48]. While the approximately four minute increase in MVPA during the school hours among intervention children was modest, they do contribute to daily MVPA and they may help to counteract the tendency toward greater inactivity with age. If gardening is integrated within the school curriculum as a pedagogical tool, and a health strategy, more time could be spent gardening and engaging in garden-based lessons, possibly yielding a stronger effect. Changes in other accelerometry-measured levels of PA during the school hours and the waking hours of the day were in the predicted direction, though not statistically significant. In addition to school gardens contributing to a reduction in usual sedentary activities and nudging children’s at-school MVPA a bit higher, our results suggest that while participating in gardening activities, children engaged in diverse physical movements and postures, using their muscles in activities such as raking, using shovels, chopping and pushing trolleys. On the other hand, children spent most their time sitting in indoor class-based lessons. Allowing children to spend more time in garden-based activities can play a role in children’s gross-motor development and strengthen muscles and bones [30].

Our study findings from the questionnaire reveal no significant impact on children’s frequency of, attitudes to and preferences in eating vegetables and fruits. Similar studies have found a positive impact of gardening on eating fruits [49,50] but findings related to impacts on eating vegetables remain contradictory and inconclusive [44,50]. However, results from qualitative data indicate some levels of acceptance on the part of children in terms of finding vegetables ‘ok’ or ‘fine’ and also some interest in trying new vegetables as opposed to not eating any vegetables. The willingness to taste new vegetables is an important objective and is the first step for the development of healthy eating behaviour among children. Our study also indicates an improvement in children’s knowledge of nutrition and plant science. Although the effect is not significant, repeated exposure to vegetables and knowledge about nutrition within the school environment through gardening may increase vegetable intake in the long run [51,52].

### 4.2. Strengths and Limitations of the Study

The study design was carefully developed to take account of known sources of bias in an experimental study. We developed the intervention taking into account the determinants of overweight/obesity and also according to guidelines for complex interventions. The use of qualitative methods help to understand the underlying reasons for any changes or no changes due to the intervention. Similar studies are mostly quantitative or purely qualitative, and very few studies explored the qualitative insights along with the quantitative evaluation. However, the study has several limitations. Firstly, the study was implemented in only one school, and hence comprised a small sample. This might explain the not significant effect of the intervention on the outcome measures. However, this study can be considered as a pilot experiment and can lead to the development of future interventions and a randomized control trial with larger samples. Secondly, the study has a short intervention timeframe and was implemented only in colder periods of the year (between September and February) when not many vegetables and fruits grow. Therefore, the activities were directed more towards conservation and maintenance of environment than growing of fruits and vegetables, hence children had limited experience of the whole growing process and were not able to harvest their grown food within that period. Although programmes as short as 10 weeks showed measurable changes in preferences for vegetables [27], an intensive programme of one whole school year could have been more effective in adequately addressing all the areas of gardening from preparation of the site to harvesting and management.

Thirdly, children on the autism spectrum and children with hearing impairment were included in the analysis. There might be debate as to whether this was appropriate, but on balance, we felt that given the inclusive nature of the study and that many children with autism spectrum disorder are in mainstream education in the UK context and they participate in the usual activities along with their peers, hence the outcomes being measured are not likely be affected by these conditions.

Fourthly, there was limited engagement of teachers in the design and planning of gardening activities and linking them with the curriculum, whereas integration in the curriculum is one of the most important success factors for school gardening programmes [26]. In addition, involvement from the Meat-Free Monday campaign was limited to one interactive session. Although online lesson plans were available on the Meat-Free Monday website, more time from teachers for direct involvement in the planning and design of activities and having some training on outdoor learning could have improved the quality of the implementation. This would also be important for later implementation and potential upscaling. Finally, one important factor possibly playing an important role in the lack of measurable effects is the fact that parents or the community were not involved in the project. Parents play a key role in children’s fruit and vegetable consumption, and the involvement of parents in school-based programmes is as important as the involvement of teachers [53] and can potentially contribute to the success of the programme [23,32].

## 5. Conclusions

Schools have been identified as a promising context for the promotion of youth PA and dietary health behaviours [54,55,56]. Thus, if gardens can be integrated more thoroughly within the school curriculum, school gardens may be one component of a school’s health promotion intervention strategy, helping children towards achieving the recommended vegetable and fruit intake and 60 min of daily MVPA. While the quantitative measures provide limited evidence of the effectiveness of the intervention, the qualitative findings indicate positive changes in many areas. Adaptations in the activities and programme can substantially contribute to increasing the effectiveness of similar projects in the future. More research with a larger sample size and longer follow-up periods is needed to examine, in more depth, the effectiveness of interventions increasing PA and promoting fruit and vegetable consumption. Recommendations are to integrate experiential outdoor learning within the national curriculum, policies should be in place for capacity building of teachers, improving their motivation and the creation of more opportunities within the school environment for such interventions to be generated and sustained. Furthermore, involvement of the parents and the community might support the effectiveness of the intervention and provide support for management and maintenance of the garden area; hence, a community-based ‘whole-school approach’ could be the key for success and sustenance of such interventions.

## Figures and Tables

**Table 1:** Background of children of the primary school based on the deprivation index (Source: Primary School).

Deprivation Index	Percentage of Children
0%−10% most deprived	4.2%
10%−20%	13.6%
20%−30%	18.4%
30%−40%	4.6%
40%−50%	10%
50%−60%	5.9%
60%−70%	16.9%
70%−80%	4%
80%−90%	0.8%
90%−100% least deprived	1.3%

**Table 2:** Questionnaire items and response scale.

Category Score (Total Items)	Items in Each Score	Number of Items	Response
Attitude			
Fruit (4)	With regards to fruit, agreement with: makes me feel healthy, tastes good, easy snack, I like tasting new fruits	4	Likert scale (1 to 5)
Vegetable (4)	With regards to vegetables, agreement with: makes me feel healthy, tastes good, I like tasting new vegetables, easy to prepare	4	Likert scale (1 to 5)
Frequency			
Fruit (1)	Number of servings of fruit consumed by you each day	1	Select from: none, 1 a day, 2–3 a day, 4−5 a day, 6 or more per day
Vegetables (1)	Number of servings of vegetables consumed by you each day	1	Select from: none, 1 a day, 2–3 a day, 4−5 a day, 6 or more per day
Preferences			
Fruit (19)	How much you like the fruits in the picture (19 fruit items that are easily available in the UK)	19	Select from: I like it a lot, I like it, It’s ok, I don’t like it, I don’t like it at all, I have never tried it and I don’t know what it is
Vegetable (19)	How much you like the vegetables in the picture (19 vegetables items that are easily available in the UK)	19	Select from: I like it a lot, I like it, It’s ok, I don’t like it, I don’t like it at all, I have never tried it and I don’t know what it is
Knowledge of plant science and nutrition			
Knowledge (7)	7 questions on what people and plants need to live, which nutrient supplies energy, which part of a the plant we eat when eating broccoli, which nutrient do we want to see on a food label, which part of the plant uses the sun’s energy, which item is not an ingredient for making compost, and which part of the plant pulls water and other nutrients from the soil	7	Select one from four options

**Table 3:** Baseline characteristics of participants.

Measures	Total	Intervention Group (IG) Mean (SD)	Control Group (CG) Mean (SD)	*p*-Value for Difference between IG and CG
Age in years	8.92 (1.20)	9.07 (0.25)	9.07 (0.25)	0.97
Sex in % girls	39.0%	40%	37.9%	0.87
Sedentary Behaviour				
SB (900−1500), minutes	244.01 (27.51)	252.72 (32.39)	234.45 (18.23)	0.042 *
SB (600−2400), minutes	839.31 (61.75)	858.91 (73.15)	825.55 (50.28)	0.137
Physical Activity				
LPA (900−1500), minutes	79.58 (16.96)	73.82 (19.72)	85.56 (11.32)	0.033 *
MVPA (900−1500), minutes	36.40 (13.25)	33.46 (14.49)	39.99 (11.49)	0.135
LPA (600−2400), minutes	168.55 (41.91)	153.83 (49.35)	178.31 (33.32)	0.105
MVPA (600−2400), minutes	73.33 (28.37)	71.067 (31.37)	76.140 (26.15)	0.594
Healthy eating				
Daily vegetable consumption	2.88 (1.21)	3.07 (1.15)	2.69 (1.26)	0.24
Daily fruit consumption	3.56 (1.20)	3.18 (1.15)	3.93 (1.13)	0.016 *
Attitude to eating vegetables	13.74 (3.33)	14.18 (3.69)	13.31 (2.94)	0.33
Attitude to eating fruits	17.30 (2.68)	17.04 (2.94)	17.55 (2.44)	0.47
Preferences of vegetable	84.26 (22.08)	84.71 (22.09)	83.83 (22.45)	0.88
Preferences of fruit	106.51 (21.24)	102.82 (23.17)	110.07 (18.91)	0.20
Knowledge of nutrition				
Knowledge of nutrition and plant science	3.05 (1.58)	2.68 (1.39)	3.41 (1.70)	0.08

* *p* < 0.05 indicates statistical significance; 900−1500 = School hours from 9 am to 3 pm; 600−2400 = Waking hours from 6 am to 12 am; SB = Sedentary Behaviour; LPA = Light Physical Activity; MVPA = Moderate to Vigorous Physical Activity; SD = Standard Deviation.

**Table 4:** Mean and standard deviation of the follow-up measures.

	Intervention Group (IG)	Control Group (CG)	*p*-Value for Difference between IG and CG
	Mean (SD)	Mean (SD)	
Sedentary Behaviour			
SB (900−1500), minutes	248.97 (62.11)	244.29 (17.90)	0.829
SB (600−2400), minutes	871.46 (93.42)	848.11 (32.76)	0.508
Physical Activity			
LPA (900−1500), minutes	69.97 (30.55)	84.99 (11.32)	0.173
MVPA (900−1500), minutes	29.44 (17.55)	30.71 (8.88)	0.842
LPA (600−2400), minutes	141.90 (69.65)	172.99(23.29)	0.244
MVPA (600−2400), minutes	64.56 (27.51)	60.00 (10.84)	0.640
Healthy Eating			
Daily vegetable consumption	2.88 (1.09)	2.90 (1.14)	0.346
Daily fruit consumption	3.35 (1.07)	3.55 (1.02)	0.728
Attitude to eating vegetables	13.07 (4.76)	14.45 (3.35)	0.085
Attitude to eating fruits	16.61 (5.07)	17.45 (2.63)	0.480
Preferences of vegetable	77.96 (29.82)	86.55 (17.66)	0.078
Preferences of fruit	97.79 (33.05)	110.24 (17.49)	0.229
Knowledge of nutrition			
Knowledge of nutrition and plant science	2.79 (1.75)	3.24 (1.76)	0.681

**Table 5:** Mean change in physical activity (minutes) over time.

Mean Change in PA	IG			CG			*p* Value
	N	Mean	SD	N	Mean	SD	
SB (900−1500)	12	−13.0388	64.73694	7	6.5423	17.21601	0.449
LPA (900−1500)	11	−4.2775	24.50218	7	−1.5536	13.02090	0.791
MVPA (900−1500)	11	2.3062	20.28664	7	−4.9887	7.18188	0.378
SB (600−2400)	8	−4.4342	34.05974	7	15.8296	23.07180	0.207
LPA (600−2400)	8	−2.0128	28.46088	7	−8.8024	19.49796	0.605
MVPA (600−2400)	11	−3.5358	45.18339	7	−7.0268	10.76162	0.845
